# Current Challenges in Cancer Immunotherapy: Multimodal Approaches to Improve Efficacy and Patient Response Rates

**DOI:** 10.1155/2019/4508794

**Published:** 2019-02-28

**Authors:** Manpreet Sambi, Leila Bagheri, Myron R. Szewczuk

**Affiliations:** Department of Biomedical and Molecular Sciences, Queen's University, Kingston, ON, Canada

## Abstract

Cancer immunotherapy is a promising innovative treatment for many forms of cancer, particularly melanoma. Although immunotherapy has been shown to be efficacious, patient response rates vary and, more often than not, only a small subset of the patients within a large cohort respond favourably to the treatment. This issue is particularly concerning and becomes a challenge of immunotherapy to improve the effectiveness and patient response rates. Here, we review the specific types of available immunotherapy options, their proposed mechanism(s) of action, and the reasons why the patient response to this treatment is variable. The potential favourable options to improve response rates to immunotherapy will be discussed with an emphasis on adopting a multimodal approach on the novel role that the gut microbiota may play in modulating the efficacy of cancer immunotherapy.

## 1. Introduction

Cancer immunotherapy was voted “breakthrough of the year” by Science in 2013 primarily due to the success rates observed at the clinical level as well as the simple yet elegant approach of this treatment [1]. However, patients that are treated with immunotherapy have shown varying response rates among cancers and within cohorts with the same malignancy [2]. Varying response rates concerning this type of therapy may be attributed to the specificity involved in eliciting an immune response, overcoming the mechanisms that cancer cells employ to evade immune surveillance, and ensuring that the activated immune cells have access to the malignant tissues. There are several ways that the response rates can be improved including, but not limited to, identifying more specific biomarkers and immune checkpoint inhibitors. Also, better predictive tools and assays can identify patients that will best respond to immunotherapy. Conceptually, this treatment approach had existed since the late 1800s but was archived as “ineffective” when radiation and chemotherapy became the standard of care for many types of cancer [3]. Currently, immunotherapy is one of the most studied forms of cancer therapy in addition to supplemental chemotherapy. The approach to cancer immunotherapy involves harnessing the specificity and killing mechanisms of the immune system to target and extirpate malignant cells.

## 2. Anticancer Immunity and Immune Evasion Mechanisms

Normal anticancer immunity involves identifying and clearing early malignant cells that express tumor-associated antigens (TAAs). TAAs are presented in complex with human leukocyte antigens (HLA) on the surface of tumor cells [3]. A complex system of interactions involving dendritic cells (DCs), macrophages, plasma cells, cytokines, antibodies, and helper T cells all work in tandem to prevent tumor development [4]. In order for an anticancer response to be initiated, TAAs that are presented by DCs in context of HLA class I molecules to activate CTLs and in context with HLA class II molecules to activate CD4+ helper T cells [5]. Activated CD4+ Th1 and Th2 helper T cells secrete interleukin-2 (IL2) and interferons (IFN) which in turn are involved in the activation of CTLs. The cytokines involved in this CTL activation and response are mainly produced by Th2 cells. Additional complexity is that for CTLs to identify tumor cells, the tumor cells must express TAAs on HLA class I molecules that initially generated the specificity of the CTLs [5].

During the tumor development, genetic mutations can also lead to the initiation of neoantigens that are recognizable by the immune system. However, once malignant cells are established, they are capable of evading this immune surveillance by turning off these antigens through a process called immune tolerance induction [4]. A second process known as immune evasion can occur when a tumor associates with its microenvironment to inhibit the antitumor response [4].

### 2.1. Cancer Evasion Mechanisms of Host Immune Response

Due to its high mutagenic capacity and keen survival capabilities, cancer cells use several mechanisms to evade the host immune response to reestablish their growth and continue to progress [6]. While many of these mechanisms are available for use in the “immune evasion toolbox,” only a handful are proposed to be useful at any given time during cancer progression based on the specific mechanism that is most appropriate for tumor establishment [7]. Key evasion tactics include upregulation of checkpoint receptor ligands that essentially prevent tumor-infiltrating lymphocytes (TILs) from entering the tumor mass, upregulation of immune-suppressing cells including regulatory T-cells (Tregs), or induction of the production of suppressive cytokines such as IL-10 and TGF-*β* [7]. Other specific mechanisms include downregulating the facets of the antigen presentation system [7].

The establishment of the tumor microenvironment (TME) not only allows the tumor to develop but permits it to recruit components of the host immune system. These TME components primarily act as cellular barriers to prevent any infiltration by antitumor immune cells in addition to promoting tumor growth [7, 8]. The development of a thick stromal layer surrounding the cancerous mass creates a physical barrier that is characterized by several features known to promote cancer growth, including the development of hypoxic conditions and abnormal tumor neovascularization [4]. Not only does this prevent potential immune cells from penetrating the tumor mass but establishes the blood vessels to allow cells to metastasize to distant organs. Once established, tumors can evade the immune system until these mechanisms are overcome, namely, by immunotherapy approaches.

## 3. Individual Immunotherapy Approaches and Factors Contributing to Varied Effectiveness

**Figure 1:**
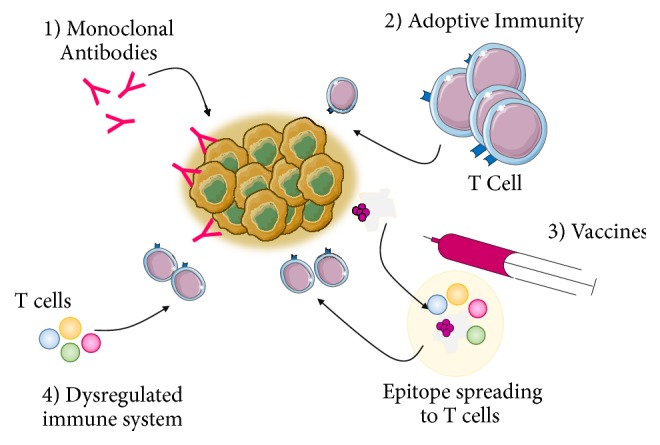
*Forms of Immunotherapy*. Currently available immunotherapy treatment options include (1) monoclonal antibodies, (1) adoptive immunotherapy, (3) vaccines, and (4) correcting a dysregulated immune system. These forms of immunotherapy are designed to either actively target a specific antigen on the tumor or enhance the host's immune system.

Five key immunotherapy modalities are now clinically approved and can be delivered to patients [9], each with varying response rates as illustrated in Figure 1. These approaches can be further classified into two general categories: active and passive immunotherapies. An additional option is the combination of them [10]. The active approach involves directing the host immune system to TAAs on the surface of tumors. These antigens can be specific proteins or carbohydrates that are exclusively expressed or overly expressed in tumor cells.

In contrast, passive immunotherapy involves enhancing the standard anticancer response by the immune system by using monoclonal antibodies, lymphocytes, and cytokines [10]. By extension, a combination therapy would involve one or more aspects of these two forms of immunotherapy. It is noteworthy that the delivery and effectiveness of immunotherapy are highly dependent on the cancer type, grade, predictive response rate, and expression of critical biomarkers [2]; however, patient response rates can still vary.

### 3.1. Monoclonal Antibodies and Their Varied Response Rates

The premise of monoclonal antibodies relies on targeting a specific antigen present on cancer cells, and it is a form of active immunity [11]. Monoclonal antibodies can either be unconjugated or be conjugated with therapeutic drugs that would produce a cytotoxic effect on cancer cells [11]. This type of immunotherapy has been used to treat many different types of cancers including breast, lymphoma, and colorectal cancer [7].

Based on the mechanism by which monoclonal antibodies exert their therapeutic effects, it is perhaps not surprising that response rates would vary. This method essentially targets a specific sequence, or epitope, of an antigen that is exclusively expressed on a tumor to induce cell death. One main reason for this variability in response rate is the fact that these monoclonal antibodies are highly specific. The “mono-” form of antibodies recognizes only one specific epitope [12] and, therefore, if there are other isoforms of the epitope due to mutations, monoclonal antibodies would be unable to recognize and bind to the antigen in question. Furthermore, the antigen that is being targeted would need to be present on the surface of cancer cells. Collectively, the specificity of monoclonal antibodies is one of the key contributors to the variation of response rates making immunotherapy ineffective.

### 3.2. Immune Checkpoint Inhibitors

Immune checkpoints function to prevent the advent of autoimmunity as a result of uncontrolled activation of T cells. Tumor cells can exploit this mechanism by deactivating tumor-infiltrating lymphocytes (TILs) and preventing them from targeting tumor cells [7]. For example, one critical immune checkpoint ligand is known as the programmed death ligand -1 or 2 (PD-L1 or PD-L2) and its receptor the programmed death-1 (PD-1) [7]. The PD-1 receptor is expressed on the surface of activated T cells and, upon binding with their ligand which is overexpressed on the surface of malignant cells, it leads the activated T cells to change their conformation to that of an inactive phenotype, rendering them ineffective [7].

**Figure 2:**
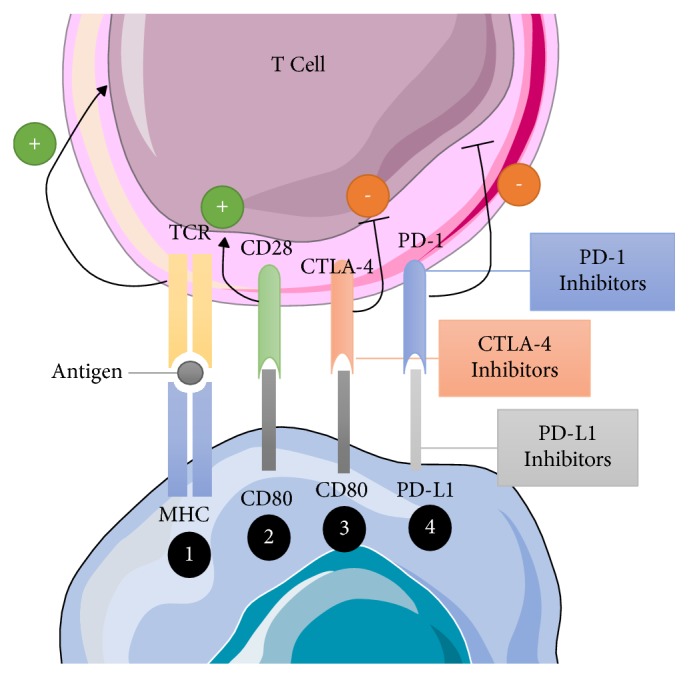
*Mechanism of Immune Checkpoint Inhibition*. MHC present antigens to the T-cell receptor in order to active T cells (1). Through interactions with the CD80 on tumor cells and the CD28 on T cells, T cells can be deactivated (2). Additionally, CTLA-4 competes with CD80 to deactivate T cells as well (3). Lastly, PD-L1 binds to the PD-1 receptor on T cells to deactivate T cells (4). Tumor cells employ the use of these mechanisms in order to prevent T cells from clearing malignant cells. By using inhibitors that prevent this interaction from occurring, T-cells remain active after identifying tumor cells and can clear them from the host.* Abbreviations:* CD; cluster of differentiation, CTLA-4; cytotoxic T-lymphocyte associated antigen-4, MHC; major histocompatibility complex, PD-1; programmed death-1, PD-L1; programmed death ligand-1, TCR; T-cell receptor.

Immune checkpoint inhibitors are a variant of monoclonal antibody immunotherapy that can block immune checkpoint receptors to allow T cells to be activated and clear tumor cells. This type of approach represents a form of passive immunity, which is designed to enhance the effectiveness of the immune system. Two currently approved checkpoint inhibitors are anti-PD-L1 and anti-cytotoxic T-lymphocyte associated antigen-4 (CTLA-4) [4, 5] as depicted in Figure 2. These two forms of immune checkpoint blockade inhibitors have been successful in several cancers including metastatic melanoma [4]. The mechanism of action of CTLA-4 involves typically competing with the ligands CD80 and CD86 to bind to CD28 in order to deactivate T cells [3]. By preventing CTLA-4 from inactivating T cells, the proposed mechanism of action that results is that T cell activity is enhanced and at the same time Treg activity is diminished [3].

Similarly, blocking PD-1 leads to a response where Tregs are deactivated and activating antitumor T-cells. Unlike CTLA-4, the PD-1 expression is on tumor cells and other healthy cells throughout the body, a large portion of which are myeloid cells in the TME [3]. This approach is far more effective when initiating an immune response. However, response rates to this immunotherapy can vary among these cancers and within tumor cohorts of the same cancer type [2].

Recent reports have implicated the gut microbiota in modulating patient response to anti-PD-1 immunotherapy in patients presenting with melanoma [13]. The basis of the report is on the clinical results of a patient previously treated with antibiotics and had a weaker response rate to PD-1 inhibition when compared to those that had not been administered antibiotics [14]. This particular study investigated the effects of gut microbiota diversity and composition on the efficacy of PD-1 and added to a growing body of research implicating the microbiota on yet another aspect of cancer treatment.

More specifically, the results from the study showed that individuals who had a more diverse microbiota composition responded better to immunotherapy as evidenced by tumor shrinkage compared with individuals with a less diverse composition. Additionally, cancer patients that responded to immunotherapy had an increase in antitumor killer T cells and the difference in killer T cell quantity was correlated to the presence of species within the* Faecalibacterium* and* Clostridiales *phyla [13]. This evidence is suggestive of certain resident bacteria contributing to a positive response rate to immunotherapies while others may dampen or render it ineffective. Because each individual's microbiome is as unique as a fingerprint, it is logical that immunotherapy response rates may differ due to the composition of the gut at the time of immunotherapy.

### 3.3. Cytokines

Under normal immune responses, cytokines directly influence immunity, where they act to enhance or inhibit the effector cellular protein components of the immune system [4]. Although other proteins modulate the immune response, for simplicity, all proteins involved in immune modulatory activities will be broadly defined as cytokines throughout this report. As such, another mode of immunotherapy involves systemically infusing specific cytokines to enhance the immune response [4]. Currently, IFN-*α* and IL-2 are administered during cancer treatment [5]. More specifically, IFN-*α* has been characterized as an immune stimulator through the activation of DC and promotion of antigen presentation to elicit an immune response, enhancing the Th1 response, CTL activity, and the cytotoxic effects of NK cells [5]. IFN-*α* is also administered in combination with cancer vaccines to enhance therapeutic effectiveness. Similarly, IL-2 has been implicated in broadly enhancing the antitumor effects of the immune system by increasing the activity of T cells, specifically tumor infiltrating cells and promoting NK cell activity [4].

While both of these types of cytokines have been used to enhance the immune system, one key caveat is that the patient would need to have a relatively robust immune system in order for these therapies to be effective. This caveat could contribute to the variation in response rates. As such, cytokines are usually used in combination with other forms of immunotherapy [4].

### 3.4. Cancer Vaccines

**Figure 3:**
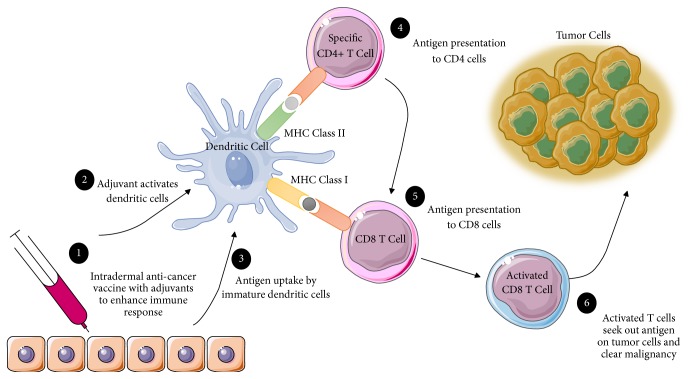
*Mechanism of Action of Cancer Vaccines*. As illustrated, cancer vaccines are administered through an intradermal injection (1) with adjuvants that activate dendritic cells (2). Immature dendritic cells take up the antigen; typically this antigen is uniquely expressed on tumor cells (3) and presents the antigen to CD4 cells (4) and CD8 cells (5). CD8 cells are then activated to seek out the antigen on the surface of tumor cells (6).* Abbreviations.* CD: a cluster of differentiation and MHC: major histocompatibility complex.

Similar to monoclonal antibody therapies, cancer vaccines are currently available as an immunotherapeutic treatment. This approach involves the conventional vaccination methods to induce an immune response. In brief, cancer vaccines that contain whole or fragments of cancer cells or antigens are designed to stimulate an immune response [11] as illustrated in Figure 3.

One main factor that contributes to differences in patient response rates to cancer vaccines is the specificity of the vaccine and whether the commercial form of the vaccine is produced with the same antigen present in the patient's tumor for the immune system to identify its presence in the body. For example, peptide-based vaccines are designed to respond to one tumor antigen in complex with its HLA [5] and thus would need to be administered to a minimal subset of patients that present this antigen. Another approach is immune- or dendritic cell-based vaccines which have shown promise in castration-resistant prostate cancer [5]. This approach involves extracting antigen-presenting cells (APCs), such as DCs, and activating them to the PA2024 prostate tumor antigen and then reintroducing the activated cells into the patient to specifically target prostate cancer cells expressing the antigen. This specific vaccine is made up of prostatic acid phosphatase (PAP), which is expressed in 95% of prostate cancers. PA2024 is delivered with granulocyte-macrophage-colony-stimulating factor (GM-CSF) in order to be taken up by APCs to active the host's T cells and, moreover, direct them to target exposed PAP on prostate cancer cells. A similar approach is applied to ovarian cancers which express the TAA CA-125 to activate DCs similarly as outlined for the prostate cancer studies. This approach assumes that a tumor is relatively homogenous and a large portion of the expressed antigen is presented to APCs. As such, this delivery strategy would significantly contribute to the efficacy of immunotherapies. Tumor heterogeneity is an issue that many cancer therapy strategies fail to consider when designing cancer vaccines. The approach requires a multimodal therapy to overcome tumor heterogeneity and is discussed in detail below.

### 3.5. Cell-Based Immunotherapy

Cell-based immunotherapy is a T-cell therapy that involves transferring natural T cells or genetically modified T cells that have been expanded* ex vivo* to target tumor antigens [7, 11] specifically. For example, these tumor antigens may include mutant proteins, tissue differentiation antigens, and vascular antigens, to name a few [11]. Once infused into the patient, cytokines such as IL-2 are delivered in combination with the therapy to enhance the effects of these activated T cells [4].

One of the most promising cell-based immunotherapies is chimeric antigen receptor T therapy (CAR-T). This approach to immunotherapy involves genetically engineering T cells* ex vivo* to enhance their specificity and antitumor mechanism of action [4]. CAR-T cells are produced* ex vivo* and reintroduced into the patient to target cancer cells in a non-HLA dependent manner specifically. This therapy can circumvent immune evasion by cancer cells that preferentially lose their HLA molecules [4]. The premise of this immune therapy requires a tumor to express “chimeric-like” antigen in order to elicit a cytotoxic response.

## 4. General Variation in Response Rates of Immunotherapy 

In the preceding sections, several forms of immunotherapies are currently available for patients; however, not all patients exhibit the same response rates. The variation of response rates reflects the therapeutic mechanism that is unique to each type of immunotherapy. In addition to the therapy-specific issues, other challenges contribute to the effectiveness of immunotherapy.

### 4.1. Pathophysiology and Tumor Microenvironment Affects Immunotherapy Access

Other components of the TME, such as tumor-associated macrophages (TAMs) and cytokines released to promote an immunosuppressive environment, also contribute to the efficacy of immunotherapy. This microenvironment makes delivery of therapeutic agents challenging, particularly concerning immunotherapy because they involve reactivating the immune system to penetrate the tumor. For example, pancreatic cancer is characterized by the development of an inflammation-driven desmoplasia, which forms a thick stromal microenvironment [15]. Therefore, not only would this barrier environment need to be disabled, but the inability of vaccines or activated T cells to penetrate the thick stromal layer would negatively affect response rates.

A recently discovered component of the microenvironment is the presence of bacteria inside pancreatic tumors that were shown to metabolize, inactivate, and potentially confer resistance to chemotherapy [16]. A similar process could potentially contribute to the varied effectiveness of immunotherapies and why response rates vary among patients with similar tumor profiles.

Lastly, perhaps the most crucial driver of differential response rates seen in cancer patients is the blood vessels and lymphatic vessels that feed into the regions surrounding the tumor. For example, mucosal tissues such as the lungs and the gastrointestinal tract are in direct contact with lymph and blood vessels and thus allow immunotherapy regimens to exert their therapeutic effects. In tissues where the TME has established minimal interaction with the surrounding physical barriers, it would be exceedingly difficult for an improvement to be observed and thus it represents another contributing factor in response rates seen in patients.

### 4.2. Development of Resistance

As with any therapy, the development of cancer resistance to the treatment is predictable; it is relatively inevitable given the highly proliferative nature of cancer cells. Immunotherapy is no different to any of the other cancer therapies. Acquired resistance in cancer cells is related to the plasticity of response mechanisms that allow cancer cells to alter their genetic pathways to compensate for changes in their immediate environment [7]. Some of these changes include epigenetic modifications and reactivation of alternative pathways [6].

One study investigated the genetic modifications in a tumor that allowed for the acquisition of resistance to the immune checkpoint inhibitor pembrolizumab [17]. Results from this study revealed that two patients had mutations in the* JAK1* or* JAK2* genes leading to a disruption in INF*γ* signalling and thereby reducing the expression of genes involved in T-cell mediated elimination of cancer cells. The genetic profile of a third patient showed a mutation in the* B2M* gene instead, which has properties in recognizing and clearing cancer cells. Based on these results, a key facet of individual response rates is related to the mutagenic nature of the type and stage of cancer and whether mutations in specific genes have taken place that renders immunotherapy ineffective.

### 4.3. Competency and Diversity of Individual Immune System

Given that immunotherapies involve the activation and amplification of the immune system, it is perhaps not surprising that differential response rates can also be the result of individual immune competency and diversity. Concerning competency, because immunotherapy is not considered a viable first-line treatment, many patients are either treated with chemo- or radiotherapy in combination with or before the administration of immunotherapy. The delivery of chemotherapy effectively reduces the competency of the immune system and would, therefore, affect patient response rates depending on the type of chemotherapy that was delivered. For example, if highly toxic chemotherapy is delivered to a patient presenting with an advanced stage of cancer and subsequently followed up with an immunotherapy, this could theoretically lead to reduced response rates as the immune system would have been compromised. Concerning immune diversity, recent reports have attributed the overall effectiveness of immunotherapy to the diversity of the HLA class I. These class-I molecules generally present intracellular processed proteins to CD8+ killer T cells, which specifically target cancerous cells expressing these processed tumor proteins [18]. Although this study considered the effectiveness of immune checkpoint blockage through anti-PD-1 or anti-CTLA-4 [19], the results can be translated to other forms of immunotherapy that involve antigen presentation like CAR-T therapy and cancer vaccines. Here, a greater diversity of HLA class I locus correlated with an overall survival following treatment [19]. The greater diversity of HLA class I molecules would be associated with an increased number of tumor antigens that could be presented thus leading to increased survivorship following therapy. Differences in HLA diversity could lead to differential response rates of immune checkpoint blockade therapies as well as other immunotherapies. However, this particular aspect of the immune system would be more relevant as a novel predictive tool and will be discussed in detail below.

### 4.4. The Composition of Gut Microbiota

The gut microbiota is composed of trillions of bacteria, viruses, and fungi that colonize the human intestine beginning at birth and acts as a natural defensive barrier to infection. It is like an organ influencing virtually every vital bodily function [20, 21]. Recently the microbiota has been proposed to influence the efficacy of chemotherapies [22] and immunotherapies [13, 23], and this is an essential factor that may contribute to the variation in the effectiveness of immunotherapies as a whole. Although the studies have established a link between the gut microbiome and how it modulates response to immune checkpoint inhibitors, conceptually, this can also be applied to other forms of immunotherapy.

For example, the gastrointestinal tract consists of the gut microbiome and the mucosal immune system that are unique to the GI tract, and it has also been shown to contribute to a host's overall immunity [24]. Since many forms of immunotherapy are designed to reactivate the immune system or enhance its effects, the diversity and health of a patient's immune system would consequently correlate with the effectiveness of immunotherapy. By extension, therefore, the positive contribution of the gut microbiota in maintaining a healthy immune system would determine the effectiveness of immunotherapy.

## 5. Future Directions of Improving Response Rates to Immunotherapy

Although several critical mitigating factors contribute to the decreased effectiveness of immunotherapy, there are several areas in which additional research is currently being done that could improve response rates of cancer patients to therapy.

### 5.1. Identification of Additional Biomarkers

More conserved biomarkers that are expressed on the surface of tumor cells need to be discovered so that immunotherapies can be applied to a broader demographic of patients [2]. The goal of active immunotherapy is to target a specific sequence that is exclusively expressed on tumor cells, which are known as “neoantigens” or tumor-specific antigens (TSA) [25]. However, many of the antigens that are expressed on tumors are also expressed on healthy cells, which would render any therapy that has a nontumor specific antigen cytotoxic to healthy cells. Identifying TSA target for immunotherapy would likely yield increased effectiveness of treatment outcomes with minimal damage to healthy cells. One example of a potential target is the cancer testis antigens (CTAs) which are expressed more readily on cancer cells as opposed to healthy cells [2]. An additional facet of these antigens is that they elicit a robust immune response. CTAs are also expressed by cancer stem cells which are a very elusive subpopulation of the tumor that contributes to its ability to self-renew indefinitely even following therapeutic intervention. Therefore, identification of additional markers would help to circumvent the challenges posed by tumor heterogeneity because the probability of targeting more than one type of cell would be increased if the host immune cells are “taught” to recognize multiple types of antigens and launch a robust attack on the whole tumor.

### 5.2. Overcoming Resistance to Immunotherapies

One advancement of overcoming resistance to immunotherapy is the use of combination immunotherapy [17] or multimodal approaches. This approach would effectively increase the probability of antigens that are targeted by the immunotherapies and thus overcoming the compensatory nature of the cancer cells. An additional facet to this model could be supplemental immunotherapy using the epigenetic blockade in order to inhibit the processes that would typically regulate gene expression in response to therapy.

As such, specific epigenetic blockade regulators have shown some promise in enhancing the effectiveness of chemotherapy. For example, cancers may employ the use of DNA methyltransferases (DNMTs) for compensation purposes. DNMTs are required in order to alter the genetic profile of cancer cells in response to the changes in the surrounding environment [26]. Theoretically, DNMTs can upregulate proliferative pathways in order to compensate for the cytotoxic effects of current therapies, including immunotherapies. Because the activity of DNMTs is likely to be higher in cancer cells compared to normal cells, DNMTs could potentially be therapeutically targeted during or following current therapies to limit the compensatory activities of the cancer. Although this has not yet been tested in humans, a knockout study conducted on the DNMT, Dmnt-1 of leukemic stem cells, was able to demonstrate the validity of targeting epigenetic modulators. Knocking out Dmnt-1 shut down leukemogenesis and leukemic stem cell renewal without affecting normal hematopoiesis [27]. An* in vitro *study on the triple negative breast cancer MDA-MB-231 cell line employed the use of SAM (*S*-adenosyl-l-methionine), an inhibitor of demethylation of cells [28]. Results from this study demonstrated that blocking this activity inhibited the metastatic ability of MDA-MB-231 cells. Taken together, a method to overcome the resistance to immunotherapies could be through the use of inhibitors that can modulate the activity of DNMTs that exclusively promote genetic alterations that affect the ability of the immune system to identify and kill cancer cells.

### 5.3. Earlier Administration of Immunotherapy

Immunotherapies are traditionally given during the later stages as second-line treatment [2]. Currently, immunotherapies have not been proposed as viable first-line treatment options, which makes it exceedingly difficult for these therapies to be effective in patients whose immune system has been compromised as a result of the conventional therapies given. One solution to this approach would be to deliver immunotherapies earlier than they are currently being done in the clinic so that the host immune system can have a robust response. Because the immunotherapy is generally administered following chemotherapy, residual tumor cells may no longer have the necessary antigens that currently acquired the immunotherapeutic target.

### 5.4. Personalized Approach to Overcoming Molecular and Physical Barriers to Immunotherapy

Due to the heterogeneous nature of many tumors as well as the unique pathophysiology of the characterized different cancers, personalized care is another solution that could potentially be used to modify response rates to immunotherapy [2]. Concerning molecular barriers, immunotherapy is only efficacious in a small subset of patients [2]. One proposed reason is that the approved immunotherapy drugs are designed to be active on a wide range of cancers, assuming they express the specific molecular profile that immunotherapies can identify and subsequently target. In addition to identifying additional targetable biomarkers, implementing a personalized approach by characterizing patient-specific tumors to test for a panel of biomarkers is an important avenue to consider. Once identified, candidate biomarkers that would elicit a robust response from the immunotherapy drug could potentially improve response rates.

Concerning physical barriers such as the dense stromal and immunosuppressive microenvironment, monoclonal antibodies that specifically target and deactivate these specific components can render the microenvironment immunosuppressive. This approach was attempted in preclinical animal studies modelling pancreatic cancer to determine a method to overcome the challenges present in the tumor microenvironment. For example, the C-X-C motif chemokine receptor 2 (CXCR-2) molecule was therapeutically targeted as a mode to overcome the immunosuppressive nature of pancreatic cancer [29]. The physiological mechanism of action of CXCR-2 is to act as a homing beacon for immune cells, specifically to attract neutrophils and myeloid suppressor cells [29]. In tumors, CXCR-2 is overexpressed on immune cells found in the tumor microenvironment of pancreatic cancer.

Additionally, this expression correlated with high levels of neutrophils and myeloid-derived suppressor cells in the tumor microenvironment contributing to pancreatic cancer progression. With impaired a CXCR-2 gene, there were decreased metastases, perhaps due to active T cells that were able to invade tumors. The principal mechanism of this study is that CXCR2 regulates T-cell infiltration. Given the immunosuppressive nature of the pancreatic cancer tumor microenvironment and the role played by CXCR-2, inhibiting this molecule could have important implications for immunotherapy. Although these applications were attempted by Steele et al. [29], many animals did not survive to receive immunotherapy. Nonetheless, this represents a promising avenue to consider in overcoming the critical challenges that contribute to various response rates.

### 5.5. Accurate Prediction of Immunotherapy Effectiveness

One area of therapeutic design to supplement conventional therapies is the use of the mutational status of cancer to assess the likelihood of a positive outcome with the targeted therapy. For example, patients that present with mutant KRAS nonsmall cell lung cancer have been well documented to be unresponsive to EGFR tyrosine kinase Inhibitor (TKI) therapy [30]. A similar predictive method is used when assessing the appropriateness of using various immunotherapies; this is especially important given the specificity of immunotherapy. Current predictive tools include characterizing the levels of PDL1 expression on a tumor to determine whether the immune checkpoint inhibition would be sufficient [3]. This approach is rational because individuals with higher rates of PD-L1 expression are more responsive to treatment [3]. As outlined above, HLA class-I diversity also acts as a predictive tool that could be used as an assay system to assess the response rates of patients treated with therapies that involve antigen presentation. Furthermore, characterizing the levels of HLA class-I diversity in tumors could be used in determining the efficacy of the treatment because this variation within cancer cells has also been linked to the treatment response rates. For example, patients' tumors that lacked HLA class-I diversity were linked with decreased survival [19]. By characterizing the HLA class-I diversity of both the tumor and the patients' immune system, predictability of immunotherapy could be determined with greater accuracy.

### 5.6. Re-Educating the Gut Microbiome to Enhance Immunotherapy Effectiveness

As outlined above, some factors contribute to the efficacy of immunotherapy and the response rates as a whole. One recently uncovered facet is the gut microbiota playing a crucial role in modulating the effects of immunotherapies. Because there are specific strains of commensal bacteria that can influence the response to immunotherapy, re-educating or diversifying the gut microbiota through the use of probiotics or prebiotics before or in conjunction with immunotherapies could lead to a robust response rate.

### 5.7. Probiotics

Probiotics are microbial food supplements that improve host gut microbiota balance. Thus far, the consensus has been that probiotics can enhance the host's immune response through several mechanisms [31]. The probiotics can promote the immune function by augmenting the mucosal barrier function, decreasing the mucosal transfer of luminal organisms and metabolites to the host, increasing the mucosal antibody production, and enhancing the epithelial integrity and direct antagonism of pathogenic microorganisms [32]. It is perhaps not surprising that the gut microbiota influences patient response to chemotherapies and immunotherapies. For example, a study by Vetizou et al. [33] demonstrated a correlation between the effectiveness of cancer immunotherapies and the composition of the gut microbiome, thereby implicating a more involved role for probiotics. In the report, CTLA-4 immunotherapy was used, and the findings from both preclinical mice studies and patients demonstrated a clear relationship between the efficacy of CTLA-4 blockade dependence on the geodistribution of* B. fragilis* in the mucosal layer of the intestine and its association with* Burkholderiales. *These relationships included synergizing with TLR2/TLR4 signalling pathways.

As an extension of these findings, researchers have attempted to rebalance the gut microbiota to increase the effectiveness of anti-cancer treatments. Sivan et al. [34] demonstrated that the rate of tumor growth decreased through oral administration of the* Bifidobacteria* alone or in combination with anti-PD-L1 immunotherapy in mouse models of melanoma. These preliminary findings suggest that there is an important underlying mechanism that could increase the potency of immunotherapies. Additional sequencing of the 16S ribosomal subunit from mice that were treated with the probiotics followed by immunotherapy demonstrated that* Bifidobacteria* were associated with antitumor T-cell responses and that, in order to improve antitumor immunity, live* Bifidobacteria* may be an essential supplement to consider when treating patients with immunotherapy. Interestingly, there are standard components of commercial probiotic supplements recapitulated the same antitumor immunity effect.

### 5.8. Prebiotics

Concerning cancer treatment, chemotherapy significantly damages the intestinal microbiota by reducing the abundance of beneficial bacteria, including* Lactobacilli* and* Bifidobacteria*, while increasing potentially pathogenic bacteria (e.g.,* Clostridia* and* Enterobacteriaceae*) [34]. As a potential treatment option, prebiotics has been proposed as a supplement to repair chemotherapy-induced intestinal dysbacteriosis. The concept of using prebiotics to target and alter the composition of the gut microbiota was first suggested in 1994 [35] and may have important implications for patient response to chemotherapy and immunotherapy.

Prebiotics are nonviable and indigestible compounds which increase the quantity of specific gut microbiota including* Bifidobacteria* and* Lactobacilli* [35]. In order to be classified as a prebiotic, a compound must be indigestible and not absorbed in the small intestine. However, it must also have the capacity to rebalance the gut microbiota to that of a healthier composition in addition to being selectively fermented by beneficial bacteria in the colon [36]. The latter leads to the production of short-chain fatty acids (SCFAs) in the colon [37]. Since the prebiotics not only modulate the gut microbiota* in vitro* (e.g., by promoting the proliferation of probiotics including* Lactobacillus plantarum L12* and* Bifidobacterium pseudocatenulatum B7003*), it also can improve the function of the bowel and immune system, the bioavailability of the metabolic health, and minerals. The prebiotics diminishes the risk and severity of the inflammatory bowel diseases (IBD) as well as the bowel syndromes unusually irritable bowel syndrome (IBS) [38]. Theoretically, if prebiotics are administered to patients and subsequently lead to the expansion of beneficial bacteria, they may have a positive implication in rebalancing the gut microbiota and could prime the host to respond favourably to immunotherapies.

### 5.9. The Novel Application of Nanotechnology to Improve the Efficacy of Immunotherapy

Applications of nanotechnology have primarily focused on revolutionizing diagnosis and improving the therapy of several types of cancer. These applications have included encapsulation of drugs into nanomaterials-based carrier systems, which may overcome their inherent limitations (e.g., their hydrophobicity and short half-lives) without the adverse effects on their therapeutic outcomes. Encapsulating therapeutics into the nanoparticles allows them to pass sequential physical and biological transport obstacles and to target the tumor tissue [39–43].

Recently, these applications have been extended to nanoparticles in fine-tuning the tumor microenvironment (TME) with the intention of rebalancing this environment and allowing for immune cell and immunotherapy transport [44]. In these studies, nanoparticles loaded with drugs, immunomodulatory substances, and oligonucleotides have been able to modulate regulatory T cell (Treg) populations indirectly. This approach is particularly significant because Tregs can act as a barrier for the effectiveness of the cancer immunotherapy [45, 46] where higher levels of Tregs have been correlated with more rapid cancer progression [47]. For instance, Kwong et al. [48] prepared liposomes anchored with anti-CD137 and IL-2Fc molecules in an* in vivo* melanoma study. The results from the study demonstrated that Treg levels were indirectly reduced, which could have critical applications in enhancing the immune response without added toxicity to the patient.

Doxorubicin is a chemotherapeutic agent used to treat breast cancer [49, 50] and has been shown to cause drug resistance [51]. Thus, an ideal doxorubicin-based tool for breast cancer should simultaneously overcome the drug resistance and inhibit the tumor-induced immunoresistance and immunosuppression [52]. Kopecka et al. demonstrated that aminobisphosphonate zoledronic acid (ZA) markedly reverses chemoresistance and immunoresistance in doxorubicin-resistant cell lines in-vitro [52]. However, administration of ZA as a free drug leads to reaching low intratumor mass, because it is intensely taken by bone [53]. To overcome this problem, Kopecka et al. encapsulated ZA within the self-assembling nanoparticles. Their results showed that the encapsulating of ZA resulted in the intratumor delivery of the aminobisphosphonate [54, 55] and enhancement of antiproliferative effects against tumors implanted in the immunodeficient animals [56, 57].

Furthermore, they investigated the impact of the nanoparticles encapsulating ZA in combination with doxorubicin on chemoresistance and immunoresistance of the breast tumors implanted in the immunocompetent mice. They observed that encapsulated ZA decreased IC_50_ of doxorubicin in human as well as murine chemoresistant breast cancer cells. It also restored the doxorubicin efficacy against a chemoimmunoresistant tumor implanted in the immunocompetent mice. Base on their findings, they suggested ZA loaded-nanoparticles as an ideal approach to simultaneously overcome the chemoresistance and immunoresistance in breast tumors.

Van Woensel et al. [58] also suggested that the intranasal nanoparticle encapsulating galactin-1 could be used as valuable adjuvant therapy in order to increase the efficiency of the immune-checkpoint blockade and chemotherapy. In the study, siRNA galactin-1-loaded chitosan nanoparticles were used to sensitize glioblastoma tumor microenvironment. Importantly, they found that both myeloid suppressor cells and Treg populations have been reduced. This approach is particularly significant because Tregs can act as a barrier to the effectiveness of the cancer immunotherapy where higher levels of Tregs have been correlated with more rapid cancer progression

Kwong et al. [48] encapsulated immunoagonists including anti-CD137 and interleukin (IL)-2Fc within nanoparticles. It is noteworthy that inflammatory toxicities limited systemic administration of the free forms of those immunotherapeutic drugs. Following intratumoral injection in the melanoma model, anti-CD137 and interleukin (IL)-2Fc loaded nanoparticles diffused into the tumor parenchyma and tumor-draining lymph nodes, while they were not able to enter the systemic circulation. The latter prevented the lethal inflammatory toxicities. Their data confirmed that the growth of simultaneously established distal tumors was inhibited significantly. They proposed that anti-CD137 and interleukin (IL)-2Fc loaded nanoparticles may have a synergistic effect in combination with the administration of well-tolerated immunotherapy agents, e.g., anti-CTLA-4 or anti-PD-1 which are known to enhance tumor regression in humans.

## 6. Future Directions in Improving Immunotherapies

**Figure 4:**
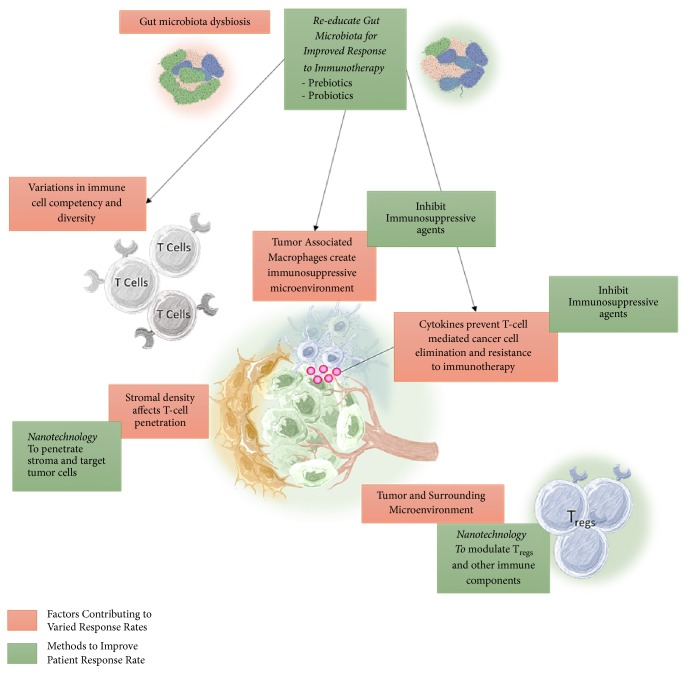
*Overview of the factors contributing to varying response rates to immunotherapy and methods to overcome these barriers*. Variations in immunotherapy response rates range from specific individual immune system diversity to the broad influence of the composition of the gut microbiota and are shown in red boxes. The proposed methods to overcome these barriers are indicated in green boxes. The gut microbiota can have overarching effects on patient response to immunotherapy due to the influence of the gut microbiota on the composition and function of the immune system.

Immunotherapy is now at the forefront of cancer treatment, but questions and challenges still remain around its efficacy, targeting, and toxicity. We have briefly detailed the latest developments in immunotherapy, including established and emerging targets and modalities, novel engineering strategies, combinations modalities, biomarkers, preclinical model approaches, strategies to mitigate toxicity, and clinical developments. Here, Figure 4 describes the overview of the factors contributing to varying response rates to immunotherapy and methods to overcome these barriers.

Examples in improving immunotherapies will come from the research on checkpoint inhibitors, adoptive T cell therapy, combinations, oncolytic viruses, manipulating the tumor microenvironment, and the gut microbiome. Technologies involved in novel gene editing with an understanding of cancer biology could unleash the full efficacy of chimeric antigen receptors T-cells (CAR-T) technology in both blood and solid tumors. Brown and Mackall [59] have recently highlighted our current understanding of resistance to CAR-T cell immunotherapy for leukaemia and lymphoma in revealing the barriers that must be addressed to increase efficacy of this novel class of therapeutics. The report identifies the key CAR-T advances and the their major problems such as the following: (a) CD19-targeted CAR-T cells produce excellent response rates in paediatric B cell acute lymphoblastic leukaemia (B-ALL) cases, but many of these patients will relapse, most often with CD19-negative leukaemia; (b) CD22-directed CAR-T cells produce high response rates in CD19 naive or resistant B-ALL but often relapse with CD22^low^ leukaemia; (c) intrinsic gene programmes of memory versus exhaustion correlate with T cell fitness and determine response to CD19-targeted CAR-T cells in chronic lymphocytic leukaemia (CLL); and (d) loss of Tet methylcytosine dioxygenase 2 (TET2), an epigenetic modulator, prevented terminal T cell differentiation and enabled the progeny of a single CD8+ CAR-T cells towards complete remission in a patient with CLL.

The development of adoptive cell therapies across a wide range of indications includes CAR-T, T cell receptors (TCR), tumor infiltrating lymphocytes (TIL), and NK cells as well as new strategies for commercialization. The immunotherapy industry is currently dominated by antagonist antibodies such as PD-1 and CTLA-4. However, it is clear that antagonists alone are not enough to elicit good response rates in the majority of patients. Hence, there are latest developments in agonist immunotherapy with a rising interest in agonist targets including TNF receptors, inducible co-stimulator (ICOS), type 2 transmembrane glycoprotein receptor belonging to the TNF superfamily and expressed on activated T Lymphocytes (4 -1BB), Toll-like receptors, stimulator of interferon genes (STING), and V-domain Ig suppressor of T cell activation (VISTA). VISTA is a type I transmembrane protein that antagonizes the programmed death-ligands 1 and 2 (PD-L1 and PD-L2); it is produced at high levels in TILs, such as myeloid-derived suppressor cells and Tregs and its blockade with an antibody results in delayed tumor growth in mouse models of melanoma [60] and squamous cell carcinoma [61]. The review by Li et al. [62] discusses the antitumor properties of TLRs, RIG-I-like receptors (RLRs), and STING-mediated innate immune pathways, in addition to the promising innate immune targets for potential application in cancer immunotherapy.

Gao et al. [63] have provided evidence to suggest that an increase in immune cell infiltration may be insufficient to generate antitumor responses. Their data were the first evidence showing that VISTA is a compensatory inhibitory pathway in the clinical treatment using ipilimumab (monoclonal anti-CTLA-4) therapy. Blockade of other immune checkpoints such as PD1/PD-L1 and/or VISTA may be necessary to provide significant clinical benefit for patients with prostate cancer. Future studies will need to elucidate the role of VISTA as a potential resistance mechanism and determine whether VISTA can be targeted to improve antitumor responses in patients.

## 7. Conclusions

Cancer immunotherapy represents a new frontier in cancer therapies that have begun to show promise since their initial conceptualization. However, patient response rates continue to fluctuate for reasons that are not well understood but have been considered from multiple standpoints, including immune competency and diversity, differing antigen specificity and expression levels, and more recently the role played by the gut microbiota. An improvement in the efficacy of immunotherapies will likely involve a more personalized and multimodal approach that cannot only target specific antigens that are present on a patient's tumor but is supplemented with agents such as epigenetic inhibitors and microbiota enhancers to elicit a more robust response. Thus, the complexity of the immune system and factors contributing to its activity are not well characterized, and additional research will require transdisciplinary approaches.
